# The Early History of PKU

**DOI:** 10.3390/ijns6030059

**Published:** 2020-07-29

**Authors:** Louis I. Woolf, John Adams

**Affiliations:** 1 Department of Neurological Sciences, University of British Columbia, Vancouver, BC V6T 1Z4, Canada; lwoolf@mail.ubc.ca; 2 Canadian PKU and Allied Disorders, Toronto, ON M5A 1N1, Canada

**Keywords:** phenylketonuria, PKU, early treatment, screening

## Abstract

The story of phenylketonuria (PKU) started in 1934 with Asbjørn Følling’s examination of two mentally retarded siblings from a Norwegian family. However, if their mother had not been so persistent in her search for somebody who could give her a reason why both her children were retarded, Asbjørn Følling’s name might never have been associated with PKU and surely the history of PKU would have started differently. In the short review below, the authors give a partly personal and therefore rare account of the early history of PKU, its treatment and the start of neonatal screening. Prof. Woolf is a pioneer of both the dietary treatment of PKU and neonatal screening; Mr. Adams is a long-time advocate for PKU patient interests.

## 1. Introduction

Asbjørn Følling, a resident in paediatrics in western Norway who had trained in chemistry before studying medicine, examined two mentally retarded siblings in 1934. To reassure the mother that he had examined them thoroughly, he tested their urine for “ketone bodies”, using ferric chloride, and obtained a green colour instead of the expected purple. He correctly identified the unknown chromogen as phenylpyruvic acid. This substance had never been found in nature before. Følling concluded that it was derived from dietary phenylalanine and named the condition oligophrenia phenylpyruvica [1,2,3,4]. His discovery was quickly followed by Jervis in the USA [5,6], and in Britain by Penrose and Quastel who renamed the condition phenylketonuria [7]. Penrose, the leading British expert on PKU and the genetics of mental retardation, held that a single gene caused both the mental retardation and the abnormal chemistry as two independent effects.

## 2. The Cause of Mental Retardation in PKU

In early 1948, Horst Bickel spent a couple of weeks in my (Dr. Woolf’s) laboratory at The Hospital for Sick Children, Great Ormond Street, London (GOS) before going to Birmingham, and from time to time we visited each other over the next few years. In 1949, David Vulliamy and I found a case of PKU at GOS and wrote it up for *Archives of Disease of Childhood*. We disagreed with Penrose [8]; we suggested that the mental retardation resulted from intoxication of the brain by phenylalanine or one of its metabolites, and that the mental retardation could be relieved by reducing the concentration of phenylalanine in the blood. We (Dr. Vulliamy and Dr. Woolf) suggested three ways of doing this; Of these, only dietary restriction proved practicable. Our paper was submitted in 1949 but appeared in print in 1951. I (Dr. Woolf) do not know where it spent the intervening 14 months [9].

## 3. First Ideas of a Phenylalanine-Restricted Diet

I (Dr. Woolf) thought an artificial diet consisting of a mixture of amino acids, without phenylalanine, to replace dietary protein, together with carbohydrates, fats, vitamins, minerals etc., might be efficacious. Individual amino acids were very expensive at that time, and I abandoned the idea. Then, I went to a meeting on the microbiological assay of amino acids in proteins using a protein hydrolysate from which phenylalanine had been removed by filtration through charcoal. Before going to GOS, I had worked at a drug company making protein hydrolysates for oral or intravenous (IV) nutrition, and I knew that they were safe and could be manufactured easily and cheaply. Things came together and I resurrected the idea of dietary treatment of PKU. At GOS the suggestion floated like a lead balloon; I was told, not unkindly, that I should be devising new diagnostic tests, not dreaming up crazy treatments for conditions that everybody knew were incurable.

## 4. First Trials with a Phenylalanine-Restricted Diet

**Figure 1:**
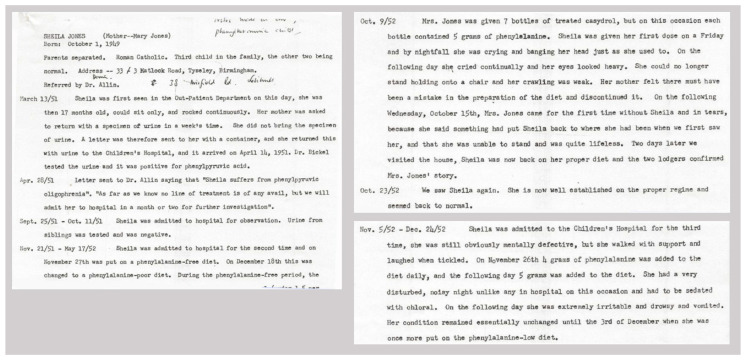
Three fragments of the original patient notes on the first phenylketonuria (PKU) patient treated, Sheila Jones. (Editorial note: as the name of this famous patient and her history are universally known, anonymization of the patient’s name in the records was considered pointless in this case and therefore omitted). Photographs are provided by Mr. Adams. Mr. Adams confirmed with Dr. John Gerrard that these case notes were made by him, including the handwriting. Gerrard confirmed that he had shared the notes with Dr. Robert Koch, and Mr. Adams found the notes when Jean Koch, the widow of Dr. Robert Koch, offered him the opportunity to peruse his files.

Bickel came to see me (Dr. Woolf) in 1952. He had a patient with PKU who was very badly deteriorated, and asked if I could suggest any treatment. I gave him full details of my proposed artificial diet (this was not as philanthropic as it sounds, since I had no hope of getting it adopted at GOS), emphasising the need to add enough phenylalanine to permit normal growth, as well as tryptophan, tyrosine, methionine (as a source of sulphur), minerals, all known vitamins, trace elements and essential fatty acids. He prepared the diet, doing much of the manual work himself, fed it to the child, and reported a dramatic improvement in her behaviour [10]. Several decades later, he confessed to me that, like my colleagues at GOS, he had thought the idea crazy, but had felt that nothing would be lost trying it. Original patient notes of the treating physician, Dr. John Gerrard, on this famous first patient treated, with details of the diet, are given in Figure 1.

When the GOS people saw Bickel’s report, it was decided to treat PKU patients with the diet but to use objective cognitive tests of the result. Ruth Griffiths joined the team in about 1958, and found a steady improvement in I.Q. over time, particularly in those not too severely brain-damaged. We also found a sudden cessation of epileptic seizures as soon as the diet was started [11].

The diet was prepared by our dietician, Frances Dillistone, using charcoal-treated casein hydrolysate purchased from Allen and Hanbury’s (a drug company and baby food manufacturer), l-tyrosine and synthetic tryptophan and methionine from a chemical manufacturer, and gluten-free wheat flour, peanut oil, sugars, calcium dihydrogen phosphate, a multi-vitamin mixture and trace elements as far as they were known at the time. Then, Allen and Hanbury’s started making the mixture for us and marketing it as “Cymogran”, leaving us to add the vitamins and minerals, and milk or cream as a source of phenylalanine. Other companies, namely, Trufood, Mead Johnson, and Scientific Hospital Supplies (Milner’s company), started manufacturing competing products (Minafen, Lofenolac, Albumaid) and yet others explored the subject. The diets were “prestige products” costing more than they could be sold for. Nevertheless, there was bitter rivalry between the companies, with one suggesting (falsely) that the other’s products lacked essential fatty acids. The protein used to make the hydrolysate was casein (Cymogran and Minafen), lactalbumin (Lofenalac) or bovine serum albumin (Albumaid). One company claimed that its competitors had chosen proteins with less than ideal amino acid compositions (pointless, since all had to add amino acids) and that its own mixture mirrored the amino acid composition of the native “ideal” protein except for phenylalanine. They forgot that, since patients with PKU could not convert phenylalanine to tyrosine, they should have added tyrosine equivalent to the sum of tyrosine plus phenylalanine in the original protein. Later they corrected this error, though without fanfare. It is ironic that the company loudest in criticism of the amino acid and essential fatty acid composition of competing products should be the only one marketing a defective product for a time.

Of course we made mistakes. At first we aimed at keeping the blood phenylalanine concentration at precisely the normal level, but it fluctuated. This meant that at times it was too low, causing the infant to go downhill. We could not at first believe how much phenylalanine (i.e., milk) a six-month old with PKU needed. We later decided to aim at a blood phenylalanine level of 1.5 to 4 mg/100 mL, i.e., 1½ to 4 times normal but still low enough to prevent mental or neurological deterioration. Methionine is a good source of cystine/cysteine in older children, but we found that neonates do not convert it efficiently and we had to add cystine or cysteine. I (Dr. Woolf) was far too obsessive in avoiding any protein except for the carefully-measured milk or cream. In retrospect, addition of more relatively low-protein natural foods would have made the diet easier to take [12].

Years later, in 1955, I found that Marvin Armstrong in Salt Lake City had reached the same conclusion about causation and treatment as myself and had treated a patient using a mixture of pure single amino acids which someone donated. This may have been before Bickel’s patient, but Armstrong did not publish until 1955 [13].

## 5. From Early Treatment to Newborn Screening

At that time, patients for treatment could be found only by testing the urine of children who presented with mental retardation at ages ranging from 1 to 12 years, and who therefore already had more-or-less severe brain damage that was only partially reversible. Then, in about 1958, the ward sisters noticed that one of our in-patients being treated with the diet was being visited by her father but not her mother. On enquiry, we were told that her mother had just given birth in another hospital. We tested the baby’s urine at age 17 days and she became the first neonate to be treated [14]. The result was excellent; she developed normal intelligence (though below that of an unaffected sibling) and eventually married and had children. This suggested that all new-born infants should be tested for PKU, but the idea met with some initial resistance from paediatricians, though later they became enthusiastic. Dr. Gibbs, Senior Medical Officer at the Public Health Department in Cardiff, was more interested and started the first large-scale screening program in Britain using urine in about 1961. We started using Phenistix on the diaper but changed to Helen Berry’s method using filter paper impregnated with urine, dried and sent to my laboratory (by that time in Oxford) [15]. We replaced testing for phenylpyruvic acid with a chromatographic method for o-hydroxyphenylacetic acid, which is more stable and provides a more sensitive test. Guthrie’s test using a blood spot proved even better (largely because the public health nurse collecting the specimen does not have to wait for the infant to pass urine), and it is a more reliable test [16]. The Guthrie blood test, like the urine test, was used for several inherited metabolic diseases.
